# Timing of Peri‐Ictal MRI Abnormalities in Status Epilepticus – One Size Does Not Fit All

**DOI:** 10.1002/ana.78076

**Published:** 2025-11-24

**Authors:** Pilar Bosque Varela, Lukas Machegger, Tanja Prüwasser, Wanda Lauth, Jürgen Steinbacher, Andreas Oellerer, Georg Zimmermann, Johannes Pfaff, Mark McCoy, Bernardo Crespo Pimentel, Markus Leitinger, Eugen Trinka, Giorgi Kuchukhidze

**Affiliations:** ^1^ Department of Neurology, Neurocritical Care and Neurorehabilitation, Christian Doppler University Hospital, Centre for Cognitive Neuroscience, Member of the European Reference Network EpiCARE Paracelsus Medical University of Salzburg Salzburg Austria; ^2^ Department of Neuroradiology, Christian Doppler University Hospital Paracelsus Medical University Salzburg Austria; ^3^ Department of Mathematics Paris‐Lodron University Salzburg Austria; ^4^ Team Biostatistics and Big Medical Data, IDA Lab Salzburg Paracelsus Medical University Salzburg Austria; ^5^ Research and Innovation Management Paracelsus Medical University Salzburg Austria; ^6^ Neuroscience Institute, Christian Doppler University Hospital, Centre for Cognitive Neuroscience Salzburg Austria; ^7^ Karl Landsteiner Institute for Clinical Neurosciences Salzburg Austria

## Abstract

**Objective:**

We aimed to investigate timing of occurrence of peri‐ictal MRI abnormalities – a potential risk biomarker of status epilepticus‐related cerebral injury (t2).

**Methods:**

This prospective study enrolled adult patients with status epilepticus and acute magnetic resonance imaging (MRI); patients with peri‐ictal MRI abnormalities underwent follow‐up MRI 4 weeks later. Predictive model, using logistic regression, integrated clinical factors (duration, semiology, and etiology of status epilepticus and the patients’ level of consciousness) to prognosticate occurrence of peri‐ictal MRI abnormalities. Cerebral injury due to status epilepticus was assessed by comparative volumetric analysis of acute and follow‐up MRIs.

**Results:**

Among 256 patients, 137 (53%) had peri‐ictal MRI abnormalities. The likelihood of their occurrence increased over time under the influence of semiology and etiology of status epilepticus as well as the patients’ level of consciousness: it was highest in non‐convulsive status epilepticus caused by acute primary or secondary etiologies at 10 minutes after onset in patients with stupor/coma (81–85%); it increased at 24 hours to 92%; and at 48 hours to 95%. Conversely, in alert/somnolent patients with prominent motor symptoms and acute triggering factors associated with epilepsy, the possibility of developing peri‐ictal MRI abnormalities at 24 hours was 4 to 5% and at 48 hours it was 6 to 11%. In 28 of 45 (62%) of follow‐up MRIs, structural long‐term consequences of status epilepticus were observed in the form of either cortical and hippocampal atrophy or global cerebral volume loss.

**Interpretation:**

In this novel multimodal approach based on MRI data, the patients’ level of consciousness, etiology, and duration of status epilepticus offers insights into the risk of possible brain injury in status epilepticus. ANN NEUROL 2026;99:523–534

Status epilepticus (SE) is one of the most common neurological emergencies^1^ with high mortality (up to 39%)^2^ and morbidity (including post‐SE epilepsy).^3^, ^4^ Despite major advances in treatment during the early phases of SE, approximately 50% of patients do not respond to the first line therapies and may, therefore, develop SE‐induced brain injury or alteration of cerebral networks.^5^ SE is currently defined by the International League Against Epilepsy (ILAE) as: “a condition resulting either from the failure of the mechanisms responsible for seizure termination or from the initiation of mechanisms, which lead to abnormally, prolonged seizures (after time point t1). It is a condition which can have long‐term consequences (after time point t2), including neuronal death, neuronal injury, and alteration of neuronal networks, depending on the type and duration of seizures.”^5^ At the time when this definition was conceived,^6^, ^7^ there were only limited experimental data from nonhuman primates and rodents^8^, ^9^ and only few clinical observations^10^ for setting the time point t2. According to the current ILAE definition of SE, t2 for tonic–clonic SE is 30 minutes and for focal SE with impaired consciousness it is over 60 minutes.^5^ For other types of SE, t2 is largely unknown. The etiology of SE is broadly categorized into acute‐, remote‐, or progressive symptomatic SE within electro‐clinical epilepsy syndromes, and cryptogenic SE (ie, without identifiable cause).^5^ Recent data suggest that a further subdivision of acute causes of SE can explain the broad variability of outcomes in the category of symptomatic SE.^11^

Peri‐ictal MRI abnormalities (PMAs) are frequently seen in patients with SE,^12^, ^13^, ^14^ across various magnetic resonance imaging (MRI) sequences, including diffusion weighted imaging (DWI), fluid‐attenuated inversion recovery (FLAIR), arterial spin labeling (ASL), or perfusion with contrast substance.^12^ PMA can be reversible or irreversible.^15^, ^16^, ^17^

Our study aimed to further delineate the concept of t2 by using human MRI data. We considered PMA a risk marker of long‐term consequences due to SE – t2. We hypothesized that longer duration of SE would increase the likelihood of developing PMA and that other factors such as level of consciousness, semiology, and etiology of an SE may influence the timing of PMA occurrence and the possibility of developing brain injury.

We anticipated that patients with diminished level of consciousness and acute primary central nervous system (CNS) etiologies would develop PMA earlier as opposed to those with preserved level of consciousness or other etiologies such as withdrawal of anti‐seizure medication (ASM).

## Materials and Methods

### Study Design

In this prospective, single‐center cohort study, adult patients (≥ 18 years old) with possible or definitive SE who underwent an MRI in an acute setting were recruited at the Department of Neurology and the Intensive Care Unit of the Department of Neurosurgery, Christian Doppler University Hospital, Paracelsus Medical University of Salzburg, Austria, between February 2019 and December 2023. All MRIs were performed at the Department of Neuroradiology of the same institution on a 3T machine, Achieva dStream (Philips Medical Systems, Best, The Netherlands).

All patients with SE underwent an MRI with a standard protocol described in the Supplementary Material (Appendix S1). Forty‐seven individuals did not undergo the ASL sequence.

### Participants, Definitions, and Categorizations

We prospectively enrolled 619 patients with an electro‐clinical diagnosis of SE. Among them, only 367 patients (59%) underwent an MRI in an acute setting. MRI was not performed in 252 of 619 (41%) patients due to following reasons: previous episodes of SE due to incompliance (n = 90), terminal disease (n = 89), known causes of epilepsy (n = 62), implants incompatible with MRI (n = 10); and extreme scoliosis (n = 1; Appendix S2, Supplementary Material).

We defined the time of occurrence of PMA as either: (I) the time from SE onset to clinical or electroencephalogram (EEG) cessation of SE. If an MRI was performed after cessation of SE, we considered the time of SE cessation as the last time point when PMA would develop; or (II) the time from SE onset to MRI in cases of ongoing SE, that is, the latest time point when PMA would develop is the time of the MRI scan.

Thus, the following patients were included in this analysis: (I) all patients with an SE, which ceased before an MRI (irrespective whether a patient had or did not have PMA); and (II) patients with an ongoing SE at the time of the MRI who had PMA. Patients with an ongoing SE who did not have PMA at the time of MRI (29 patients), were excluded from the analysis as we could not determine whether PMA would develop after the time point when the MRI was performed.

Patients with cerebral hypoxia following cardiac arrest (25 patients) and those with unknown clinical duration of SE (52 patients) were excluded from the study. Furthermore, patients with absence SE (5 patients) were excluded due to their distinct pathophysiological characteristics. In total, 111 of 367 (30%) patients were excluded from the study and eventually the data of 256 of 367 (70%) underwent the analysis (Appendix S2, Supplementary Material; Table 1).

**TABLE 1:** Demographic and Clinical Characteristics of Patients With SE

Variables		N = 256
Age (median, IQR)		67 (55–78)
F		110 (43%)
Clinical duration of SE, hours (median, IQR)		2 (0.60–4.99)
De novo SE		165 of 256 (64%)
Semiology	SE‐PM	122 of 256 (48%)
	NCSE	110 of 256 (43%)
	SE‐PM into NCSE	24 of 256 (9%)
Etiology	Category I	18 of 256 (7%)
	Category II	78 of 256 (30%)
	Category III	160 of 256 (63%)
Level of consciousness	Alert/ somnolent	189 of 256 (74%)
	Stupor/ coma	67 of 256 (26%)
MRI performed	After SE resolution	191 of 256 (75%)
	During ongoing SE	65 of 256 (25%)
Time from SE onset to MRI, h (median, IQR)	After SE resolution	1.16 (0.50–3.36)
	During ongoing SE	3.51 (1.75–19.83)
Outcome	EMSE (median, IQR)	54 (31–77)
	STESS (median, IQR)	3 (2–4)
	30‐day mortality rate	28 of 256 (11%)

EMSE = epidemiology based mortality score in status epilepticus; Etiology I = acute‐triggering factors associated with epilepsy (TFE); Etiology II = acute primary and secondary central nervous system insults, and acute toxic; Etiology III = progressive, remote, unknown, SE in electro‐clinical syndromes; IQR = interquartile range; MRI = magnetic resonance imaging; NCSE = SE without prominent motor symptoms (non‐convulsive SE); RSE = refractory SE; SE = status epilepticus; SE‐PM to NCSE = SE with prominent motor symptoms, which developed into SE without prominent motor symptoms (non‐convulsive SE); SE‐PM = SE with prominent motor symptoms; SRSE = super‐refractory SE; STESS = Status Epilepticus Severity Score.

The median time from the presumed onset of an SE to an MRI was 21.6 hours (interquartile range [IQR] = 3.9–44.9); the median time from the hospital admission to an MRI was 19 hours (IQR = 2.8–42.5). An MRI was performed within the first 6 hours after the onset of SE in 101 of 256 (39%) patients, in an interval between 5 and 12 hours in 9 of 256 (4%) patients, between 12 and 24 hours in 53 of 256 (21%) patients, between 24 and 48 hours in 44 of 256 (17%) patients, and in 49 of 256 (19%) patients in more than 48 hours after the SE onset.

Patients were divided into 3 main categories based on their semiology, following ILAE classification^5^: (I) with prominent motor symptoms (SE‐PM), (II) without prominent motor symptoms (ie, non‐convulsive SE [NCSE]), and (III) transition from SE‐PM to NCSE. We divided patients based on the level of consciousness into 2 groups: (1) those who were alert or somnolent, and (2) those in a stupor or coma. Consciousness was assessed clinically before any treatment with ASM.

Etiology was subdivided into 3 categories based on a proposal by Lattanzi et al^11^ and on the ILAE definitions: (I) acute triggering factors associated with epilepsy (TFE); (II) acute primary and secondary CNS insults and acute toxic; and (III) other etiologies such as progressive, remote, unknown, and SE in electro‐clinical syndromes.

In this study, we chose to focus on diffusion restriction and FLAIR‐hyperintensity, which occur in patients with SE. These abnormalities may reverse in some patients or persist for days or even weeks in others, leading to subsequent irreversible changes in form of cortical atrophy or hippocampal sclerosis, as has been shown in human and animal studies.^17^, ^18^, ^19^, ^20^, ^21^

Diffusion restricted lesions and FLAIR‐hyperintensities were assessed visually by 2 independent raters (authors G.K. and L.M.) blinded to clinical data. Disagreements were resolved after consulting a third reviewer (author J.P.) and the inter‐rater agreement was calculated using Cohen's kappa coefficient.

Diffusion restricted lesions and hyperintense signal in FLAIR were attributed to PMA if they fulfilled at least one of the following criteria: (I) PMA affecting brain areas such as pulvinar of thalamus or hippocampus in combination with cortex if they did not respect vascular territories^22^, ^23^, ^24^, ^25^; (II) presence of a simultaneous hyperperfusion in ASL^26^; and (III) in case of diffusion‐restricted lesions, they were classified as PMA if the quantification revealed a signal intensity ratio lower than 1.495 for DWI and higher than 0.735 for apparent diffusion coefficient (ADC).^27^ In the quantification analysis, we used 4 b‐values for calculating the conventional ADC map, which is based on the logarithm of signal intensity of the diffusion image divided by signal intensity of the image without diffusion gradient ADC = −1/*b* ln(*S*_DWI_/*S*_0_). We compared intensities of gray values of diffusion‐restricted lesions to the healthy mirror side in DWI slices with a *b*‐value of 1,000 and in ADC maps. The size of the region of interest (ROI) was chosen to cover the maximum signal intensity of the lesion, the equal size was used for the mirror side. In case of anatomic asymmetry of brain hemispheres, the reference ROI on the healthy side was chosen in the most corresponding anatomic region. A target lesion with the maximum signal intensity in diffusion b1000 was selected for each patient. The resulting average gray value intensity of the selected area was compared between a lesion and a healthy side.

The quantification of the diffusion restricted lesions was done by drawing manually circular ROIs using IntelliSpacePortal version 10.1. The aforementioned cutoff values for distinguishing diffusion restriction between patients with SE and acute stroke were established in our previous study, where we calculated and compared ratios of signal intensity values of DWI and ADC on the lesion side to the healthy mirror side for both groups of patients (SE and acute stroke).

MRI abnormalities were not classified as PMA if they were considered as primary CNS lesions (83/256, 32%): brain tumor (n = 31), old ischemic stroke (n = 20), acute ischemic stroke (n = 14), old cerebral hemorrhage (n = 7), acute encephalitis (n = 4), acute cerebral hemorrhage (n = 3), meningioma (n = 2), multiple cavernomas (n = 1), and brain abscess (n = 1).

In 36 of 256 (14%) patients, no lesions were seen on MRI (Appendix S2, Supplementary Material).

### Follow‐Up MRIs and Volumetric Analysis

Follow‐up MRIs were performed 4 weeks following the SE only in those patients who demonstrated PMA in the initial acute MRI. Reversibility of PMA was assessed visually. Cortical thickness was measured and compared between initial and follow‐up MRIs. Hippocampal volume was calculated by comparing the volume ratios of the affected hippocampus and contralateral healthy hippocampus between initial and follow‐up MRIs.

Diffuse brain volume was assessed by measuring the ventricular‐to‐brain ratio (VBR) on T1‐weighted images of the initial MRI and a follow‐up MRI.

Voxel‐based morphometry was performed using FreeSurfer software (version 7.2.0). The VBR between the initial and follow‐up MRIs was compared to estimate diffuse brain atrophy.

The surface‐based stream in FreeSurfer involved several key steps:

1. Preprocessing: T1‐weighted images underwent motion correction and intensity normalization.
2. Skull Stripping: This step isolated the brain from non‐brain tissues.
3. Cortical Surface Reconstruction: The cortical surface was reconstructed by identifying the boundary between gray and white matter and the pial surface. This process included automated topology correction and surface deformation to achieve accurate representations of the cortical surface.
4. Cortical Parcellation: Neuroanatomic labels were assigned to each region of the cortical surface based on a probabilistic atlas. These regions were used to measure cortical thickness, calculated as the distance between the white matter surface and the pial surface. The final output provided detailed morphometric data, including cortical thickness across the entire cortex.

### Expected Bias and Ways of their Prevention

We did not expect that all patients with SE would undergo an MRI upon entering our hospital. MRI is a routine test in patients with SE as over 60% of SE cases occur without a known epilepsy diagnosis.^12^ However, one of the common causes of SE is a low level of antiepileptic drugs due to noncompliance in patients with an established diagnosis of epilepsy. Such cases are associated with a low mortality rate and usually manageable with the first line treatment of SE.^11^ These patients are not commonly admitted to the inpatient ward and are released after spending some hours in an emergency department. Such patients normally do not undergo MRI and the MRI changes due to SE are not expected. Obviously, this caused a selection bias in our study. However, we expected, based on our longstanding clinical experience and data from previous studies related to SE, that the majority of the patients with SE who will be admitted to the inpatient facility of our hospital will undergo an MRI.^12^, ^13^, ^22^, ^27^, ^28^ Hence, this study focused on those patients with SE who underwent in‐hospital treatment.

### Data Analysis

Based on the data of our cohort, which included patients with or without PMA, logistic regression models were built for predicting the probability of PMA occurrence as a risk biomarker for t2 at various time points after the onset of SE (Table 2). The SE semiology, etiology, and level of consciousness were incorporated into the model as factors, which could possibly influence the development of PMA. The time points when PMA could possibly occur were analyzed across various intervals during the progression of SE, with a specific focus on the initial 1‐hour and 5‐hour periods.

**TABLE 2:** Logistic Regression Model: Diffusion Restriction and FLAIR‐Hyperintensity

	DWI				FLAIR			
	OR	95% CI	*p*	*p* adjusted	OR	95% CI	*p*	*p* adjusted
Clinical duration	1.02	1.003–1.05	0.03	0.38	1.03	1.009–1.05	0.007	0.11
NCSE vs SE‐PM	5.63	2.88–11.46	0.00000078	**< 0.001**	4.22	2.11–8.80	0.000071	**0.001**
SE‐PM into NCSE vs SE‐PM	1.92	0.61–5.77	0.247	0.99	1.75	0.52–5.59	0.35	0.99
Etiology II vs Etiology I	10	2.22–100	0.007	0.11	7.69	1.72–100	0.01	0.21
Etiology II vs Etiology III	2.32	1.20–4.54	0.001	0.15	3.125	1.61–6.25	0.0009	**0.015**
Stupor/coma vs alert/somnolent	5.34	2.72–10.81	0.0000016	**< 0.001**	5.60	2.85–11.35	0.00000089	**< 0.001**

Statistically significant differences between the groups are marked in bold.

95% CI = confidence interval; DWI = diffusion weighted imaging; Etiology I = acute‐triggering factors associated with epilepsy (TFE); Etiology II = acute primary and secondary central nervous system insults, and acute toxic; Etiology III = progressive, remote, unknown, SE in electro‐clinical syndromes; FLAIR = fluid attenuated inversion recovery; NCSE = SE without prominent motor symptoms (non‐convulsive SE); OR = odds ratio; SE = status epilepticus; SE‐PM = SE with prominent motor symptoms.

The prediction model was utilized with respect to each MRI sequence (DWI and FLAIR).

**FIGURE 1:**
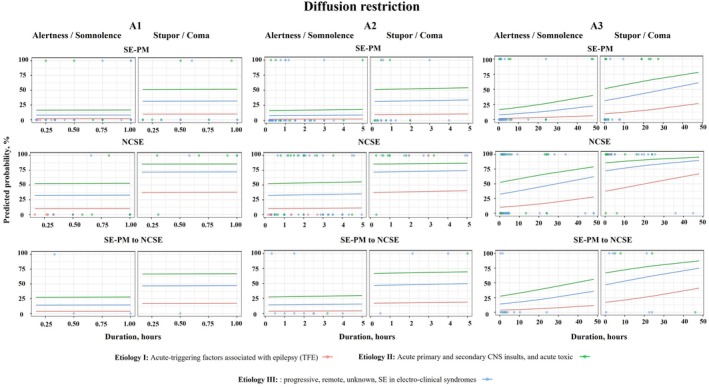
Model of predictive probabilities of diffusion restriction (A) in the time course of SE based on semiology, level of consciousness, and etiology. Different time scales have been shown on panels: A1 = first hour after the onset of SE; A2 = first 5 hours after the onset of SE; and A3 = first 50 hours after the onset of SE. The translucent points represent the real data with a possibility for each patient of either displaying PMA (100% on the plot) or not having PMA (0% on the plot). The color lines represent predicted probabilities of developing diffusion restriction in different clinical situations reflecting SE semiology, etiology, and the patient's level of consciousness. NCSE, SE without prominent motor symptoms (non‐convulsive SE), SE‐PM, SE with prominent motor symptoms. Etiology I = acute‐triggering factors associated with epilepsy (TFE); Etiology II = acute primary and secondary CNS insults, and acute toxic; Etiology III = progressive, remote, and unknown SE in electro‐clinical syndromes. CNS = central nervous system; PMA = peri‐ictal magnetic resonance imaging abnormalities; SE = status epilepticus.

**FIGURE 2:**
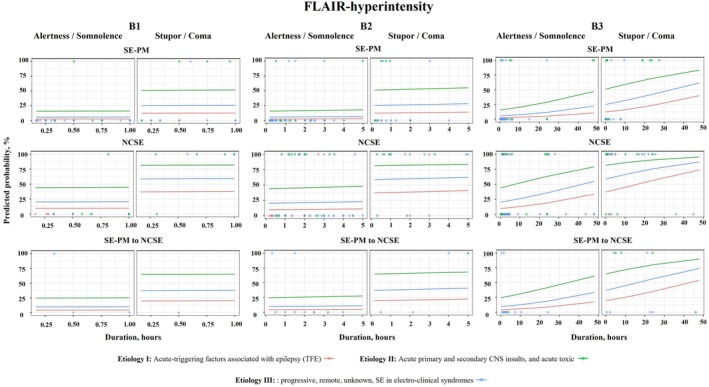
Model of predictive probabilities of FLAIR‐hyperintensity (B) in the time course of SE based on semiology, level of consciousness, and etiology. Different time scales have been shown on panels: B1 = first hour after the onset of SE; B2 = first 5 hours after the onset of SE; B3 = first 50 hours after the onset of SE. The translucent points represent the real data with a possibility for each patient of either displaying PMA (100% on the plot) or not having PMA (0% on the plot). The color lines represent predicted probabilities of developing FLAIR‐hyperintensity in different clinical situations reflecting SE semiology, etiology, and the patient's level of consciousness. NCSESE without prominent motor symptoms (non‐convulsive SE); SE‐PM SE with prominent motor symptoms. Etiology I = acute‐triggering factors associated with epilepsy (TFE); Etiology II = acute primary and secondary CNS insults, and acute toxic; Etiology III = progressive, remote, and unknown SE in electro‐clinical syndromes. CNS = central nervous system; FLAIR = fluid‐attenuated inversion recovery; PMA = peri‐ictal magnetic resonance imaging abnormalities; SE = status epilepticus.

Predicted probabilities of PMA occurrence and their possible timing in different clinical situations based on SE semiology, etiology, and level of consciousness were presented. This analysis is reflected in Figures 1 and 2: the colored circles are real patients who had either PMA (100%) or no PMA (0%); the colored lines represent the possible chances of developing PMA over time depending on etiology and semiology of SE as well as the patients’ level of consciousness. For instance, patients with acute etiologies (etiology II, green lines) in stupor or coma have greater chances of developing PMA as opposed to those patients with TFE (etiology I, red lines) with SE‐PM who were alert or somnolent.

Additionally, we assessed the timing of 25%, 50%, and 75% chances of developing PMA in different clinical situations based on SE semiology, etiology, and patients’ level of consciousness. Furthermore, an arbitrary 10‐minute cutoff was chosen for demonstrating the chances of developing PMA again depending on the clinical scenario (Table 3).

**TABLE 3:** Predictive Probabilities of Developing Peri‐Ictal MRI Abnormalities (PMA) as Surrogate Risk Marker for t2

	Chances of developing diffusion restriction				Chances of developing FLAIR hyperintensity			
	At 10 minutes after the onset of SE	25%	50%	75%	At 10 minutes after the onset of SE	25%	50%	75%
SE‐PM, Etiology I, alert/somnolent	2%	*None at 48 h*	None at 48 h	*None at 48 h*	2%	*None at 48 h*	None at 48 h	*None at 48 h*
SE‐PM, Etiology I, stupor/coma	10%	*At 45 h*	None at 48 h	*None at 48 h*	12%	*At 27 h*	None at 48 h	*None at 48 h*
SE‐PM, Etiology II, alert/somnolent	16%	*At 21 h*	None at 48 h	*None at 48 h*	16%	*At 18 h*	None at 48 h	*None at 48 h*
SE‐PM, Etiology II, stupor/coma	**51%**	***< 10 minutes***	**< 10 minutes**	*At 41 h*	**51%**	***< 10 minutes***	**< 10 minutes**	*At 32 h*
SE‐PM, Etiology III, alert/somnolent	8%	*None at 48 h*	None at 48 h	*None at 48 h*	6%	*None at 48 h*	None at 48 h	*None at 48 h*
SE‐PM, Etiology III, stupor/coma	31%	***< 10 minutes***	At 31 h	*None at 48 h*	25%	***< 10 minutes***	At 33 h	*None at 48 h*
NCSE, Etiology I, alert/somnolent	10%	*At 43 h*	None at 48 h	*None at 48 h*	10%	*At 35 h*	None at 48 h	*None at 48 h*
NCSE, Etiology I, stupor/coma	37%	***< 10 minutes***	At 20 h	*None at 48 h*	37%	***< 10 minutes***	At 16 h	*None at 48 h*
NCSE, Etiology II, alert/somnolent	**52%**	***< 10 minutes***	**< 10 minutes**	*At 39 h*	44%	***< 10 minutes***	At 7 h	*At 41 h*
NCSE, Etiology II, stupor/coma	**85%**	***< 10 minutes***	**< 10 minutes**	***< 10 minutes***	**81%**	***< 10 minutes***	**< 10 minutes**	***< 10 minutes***
NCSE, Etiology III, alert/somnolent	32%	***< 10 minutes***	At 29 h	*None at 48 h*	20%	*At 9 h*	At 42 h	*None at 48 h*
NCSE, Etiology III, stupor/coma	**72%**	***< 10 minutes***	**< 10 minutes**	*At 6 h*	**59%**	***< 10 minutes***	**< 10 minutes**	*At 23 h*
SE‐PM into NCSE, Etiology I, alert/somnolent	4%	*None at 48 h*	None at 48 h	*None at 48 h*	4%	*None at 48 h*	None at 48 h	*None at 48 h*
SE‐PM into NCSE, Etiology I, stupor/coma	17%	*At 19 h*	None at 48 h	*None at 48 h*	20%	*At 10 h*	At 43 h	*None at 48 h*
SE‐PM into NCSE, Etiology II, alert/somnolent	27%	***< 10 minutes***	At 38 h	*None at 48 h*	24%	***< 10 minutes***	At 34 h	*None at 48 h*
SE‐PM into NCSE, Etiology II, stupor/coma	**67%**	***< 10 minutes***	**< 10 minutes**	*At 16 h*	**65%**	***< 10 minutes***	**< 10 minutes**	*At 15 h*
SE‐PM into NCSE, Etiology III, alert/somnolent	14%	*At 28 h*	None at 48 h	*None at 48 h*	9%	*At 35 h*	None at 48 h	*None at 48 h*
SE‐PM into NCSE, Etiology III, stupor/coma	47%	***< 10 minutes***	At 5 h	*None at 48 h*	37%	***< 10 minutes***	At 16 h	*None at 48 h*

In bold and italic are marked the possible chances of developing either diffusion restriction or FLAIR hyperintensity within the first 10 minutes after the onset of status epilepticus.

Etiology I = acute‐triggering factors associated with epilepsy (TFE); Etiology II = acute primary and secondary central nervous system insults, and acute toxic; Etiology III = progressive, remote, unknown, SE in electro‐clinical syndromes; FLAIR = fluid attenuated inversion recovery; MRI = magnetic resonance imaging; NCSE = SE without prominent motor symptoms (non‐convulsive SE); PMA = peri‐ictal magnetic resonance imaging abnormalities; SE = status epilepticus; SE‐PM, SE with prominent motor symptoms.

Odds ratios for the development of PMA in both MRI sequences depending on clinical situations were calculated.

**FIGURE 3:**
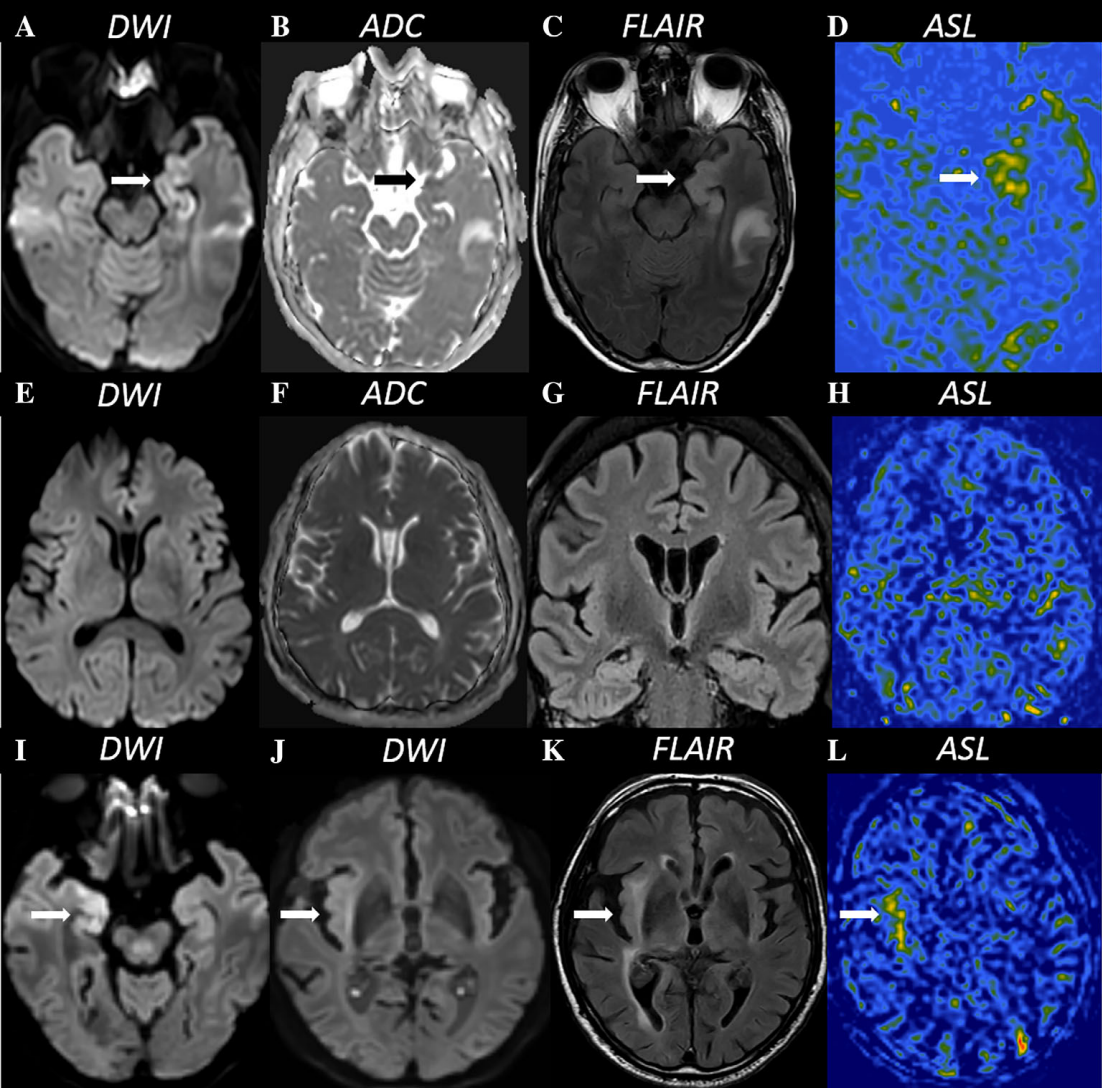
Model of predictive probabilities in clinical practice. Patient 1 (*top row* A to D) with an NCSE (aphasic SE). Level of consciousness: alert. Duration of SE = 24 hours. Etiology = acute intracranial hemorrhage (Etiology group II) = predictive probabilities of diffusion restriction – 67% and hyperintense signal in FLAIR of 63%. MRI findings: diffusion restriction in left hippocampus (*arrows*, A and B). Hyperintense signal in FLAIR in left hippocampus (*arrow*, C). Hyperperfusion in the left mesial temporal area (*arrow*, D). Patient 2 (*second row* E – H) with a SE‐PM. Level of consciousness = somnolent. Duration = 0.5 hours. Previous epilepsy history. Etiology = withdrawal of ASM (Etiology group I). Predictive probabilities of diffusion restriction – 1.95% and hyperintense signal in FLAIR – 2%. MRI findings = no PMA. Patient 3 (*third row* I–L) with an NCSE. Level of consciousness = somnolent. Etiology = remote cerebrovascular (Etiology III). Duration = > 48 hours. Predictive probabilities of diffusion restriction – 62% and hyperintense signal in FLAIR – 55%. MRI findings = diffusion restriction in the right hippocampus (*arrow*, I), right insula and claustrum (*arrow*, J). FLAIR‐hyperintensity in the right insula and claustrum (*arrow*, K), and hyperperfusion in the right insula and claustrum (*arrow*, L). ADC = apparent diffusion coefficient; ASL = arterial spin labeling; ASM = anti‐seizure medication; DWI = diffusion weighted imaging; FLAIR = fluid attenuated inversion recovery; MRI = magnetic resonance imaging; NCSE = SE without prominent motor symptoms (non‐convulsive SE); SE = status epilepticus; SE‐PM = SE with prominent motor symptoms.

This model enables the prediction of PMA in different patient groups or clinical situations. It gives a possibility of answering questions such as “what is the probability of developing diffusion restriction related to an SE in a patient with a SE‐PM in a coma and withdrawal of ASM as an etiological factor in 30 minutes after the onset of SE?” (Fig 3).

There were no missing data in the analysis.

### Statistical Analysis

Data were extracted and tabulated, and then analyzed with descriptive and inferential statistics using R software version 4.1.3 and SPSS. Categorical variables were summarized with frequencies and percentages. Continuous variables were summarized with median and IQR.

For each MRI sequence, a logistic regression model was fitted to the data using occurrence of PMA as dependent variable and semiology, etiology, and level of consciousness, as well as the time from SE onset to clinical and/or EEG cessation of SE or until an MRI was performed as independent variables. The estimated odds ratios (ORs) from the models with 95% confidence intervals (95% CIs) were reported as well as the *p* values, which were adjusted for multiplicity using the Bonferroni‐Holm method. Additionally, the models were used in order to predict an estimated probability of developing PMA (for each sequence separately) for certain clinical situations in terms of the predictor variables at some predefined time points.

### Ethical Issues

The Ethics Committee of the Region of Salzburg approved this study on human subjects (approval number 415‐E/2422).

This study was conducted based on the Strengthening the Reporting of Observational Studies in Epidemiology (STROBE) reporting guidelines.

## Results

Clinical and demographic characteristics of this cohort are summarized in Table 1. PMAs were observed in 137 of 256 (54%) patients. No significant association with PMA occurrence was found for age (*p* = 0.830) and gender (*p* = 0.899). Cohen's kappa on interrater agreement in attributing diffusion restriction and FLAIR‐hyperintensity to an SE (PMA) were 0.84 (95% CI = 0.77–0.91) and 0.90 (95% CI = 0.84–0.96) respectively, indicating almost perfect consensus. The median time from SE onset to MRI was significantly longer in patients with an ongoing SE (3.51 hours, IQR = 1.75–19.83 hours) as compared with those with resolved SE (1.16 hours, IQR = 0.5–3.36 hours, *p* < 0.001). In the subgroup of patients who underwent MRI after SE resolution, the SE duration was significantly longer in those with PMA (median = 2.5 hours, IQR = 1.00–5.04 hours) compared with those without PMA (median = 0.75 hours, IQR = 0.33–2.12 hours, *p* < 0.001).

### Diffusion Restricted Lesions

Diffusion restricted lesions were observed in 87 of 256 (34%) patients. Absolute and relative frequencies of patients showing DWI abnormalities were calculated at clinically relevant intervals from SE onset to its resolution (Supplementary Table S7). DWI abnormalities were observed in 6.5% of patients with SE duration < 30 minutes, increasing to 28% for < 5 hours, and 34% for < 30 hours. These findings suggest a progressive increase in DWI signal changes with longer SE duration, which appears to plateau beyond 30 hours.

The logistic regression model (Table 2) revealed that SE semiology and level of consciousness significantly influenced occurrence of diffusion‐restricted lesions. Specifically, NCSE was more frequently associated with diffusion restriction compared to SE‐PM (OR = 5.63, 95% CI = 2.88–11.46, *p* < 0.001, adjusted). In patients with stupor or coma, there was a higher risk of developing diffusion restriction compared with those who were alert or somnolent (OR = 5.34, 95% CI = 2.72–10.81, *p* < 0.001, adjusted).

The chances of developing diffusion restriction increased over time under the influence of SE semiology and level of consciousness. Figures 1 and 2 show that in patients with SE‐PM who were alert or somnolent and had TFE as an etiology of SE, the chances of developing diffusion restriction were the lowest as compared with other groups. In this group of patients, the chances of developing diffusion restriction at 24 hours after the onset of SE were 4%, they increased at 48 hours to 6%. As opposed to this group, the possibility of developing diffusion restriction in patients with NCSE in coma with an acute etiology (category II) were 86% already at 10 minutes. The chances increased to 92% at 24 hours and to 95% at 48 hours. Details of the prediction of diffusion restriction at different time points in relation to SE semiology, etiology, and level of consciousness are presented in Table 3 and Supplementary Tables S1 to S3.

As shown in Table 3, over 50% probability (Pr) of developing diffusion restriction already at 10 minutes after the onset of SE can be expected in the following groups of patients: (1) NCSE, etiology II, stupor/coma (Pr = 86%); (2) NCSE, etiology III, stupor/coma (Pr = 72%); (3) SE‐PM to NCSE, etiology II, stupor/coma (Pr = 67%); (4) NCSE, etiology II, alert/somnolent (Pr = 52%); and (5) SE‐PM, etiology II, stupor/coma (Pr = 51%).

In addition, Table 3 shows the 25% and 75% chances of developing diffusion restriction at different time points along with aforementioned 50% chance.

### FLAIR‐Hyperintensity

Hyperintense signal in FLAIR as PMA was observed in 75 of 256 (29%) patients. The frequency of FLAIR changes increased with SE duration, from 4.4% of patients with duration < 30 minutes to 23% for < 5 hours and 29% for < 30 hours. Compared to DWI, FLAIR abnormalities were slightly less frequent across all thresholds but showed a similar temporal pattern of gradual rise and plateau. Detailed numbers for each SE duration are shown in Supplementary Table S7.

Logistic regression analysis, as detailed in Table 2, revealed that SE semiology, level of consciousness, and certain etiologies were associated with a rate of occurrence of FLAIR‐hyperintensity. Patients with NCSE had FLAIR‐hyperintensity more frequently as compared with those with SE‐PM (OR = 4.22, 95% CI = 2.11–8.80, *p* = 0.001, adjusted). Individuals in stupor or coma demonstrated higher incidence of FLAIR‐hyperintensity as opposed to those who were alert or somnolent (OR = 5.60, 95% CI = 2.85–11.35, *p* < 0.001, adjusted). Acute etiologies (category II) were more commonly associated with FLAIR‐hyperintensity compared with other etiologies (category III, OR = 3.125, 95% CI = 1.61–6.25, *p* = 0.015, adjusted).

The likelihood of developing FLAIR‐hyperintensity increased over time under the influence of SE semiology, etiology, and level of consciousness.

As shown in Figures 1 and 2 as well as in Table 3, patients with TFE as the primary etiology (category I) who were alert or somnolent, had the lowest chances of developing FLAIR‐hyperintensity. In this group, a 50% probability of FLAIR‐hyperintensity would not be reached even after 48 hours, irrespective of semiology. Conversely, patients with acute etiologies (category II), presenting with SE‐PM, NCSE, or SE‐PM to NCSE in stupor or coma, had an estimated 50% probability of developing FLAIR‐hyperintensity within less than 10 minutes after the onset of SE. After 10 minutes, these probabilities escalated for SE‐PM and SE‐PM to NCSE to 51% and 65%, respectively. The chances of developing FLAIR‐hyperintensity were highest in patients with NCSE and acute etiologies (category II) in 10 minutes after the onset of SE in patients with stupor/coma (Pr = 81%) but also in those who would be alert/somnolent (Pr = 44%).

At least a 50% chance of developing FLAIR‐hyperintensity as early as 10 minutes after SE onset (see Table 3) may apply to the following groups: (1) NCSE, etiology II, stupor/coma (81%); (2) SE‐PM to NCSE, etiology II, stupor/coma (65%); (3) NCSE, etiology III, stupor/coma (59%), and (4) SE‐PM, etiology II, stupor/coma (51%). The details of the prediction of FLAIR‐hyperintensity at different time points in relation to SE semiology, etiology, and level of consciousness are presented in Supplementary Tables S4 to S6. In addition, Table 3 shows 25% and 75% chances of developing FLAIR‐hyperintensity at different time points along with the aforementioned 50% chance.

Figure 3 shows 3 illustrative examples of the use of the predictive model in clinical practice.

### Follow‐Up MRIs and Volumetric Analysis

In 92 of 256 (36%) patients, either diffusion restriction, FLAIR‐hyperintensity, or both at the same time were seen on the initial MRI performed in the acute phase. Follow‐up MRI with the same protocol as for the initial MRI, was performed in 45 of 92 (49%) patients 4 weeks following the SE. In 38 of 45 (84%) patients, PMAs were completely resolved on the follow‐up MRI; in 7 of 45 (16%) patients, on the contrary, PMAs were still present. In total, 28 of 45 (62%) patients developed long‐term structural consequences demonstrated by the volumetric analysis: 3 patients had local cortical atrophy, corresponding to the location of PMA; 10 patients had isolated unilateral hippocampal atrophy; 12 patients had global cerebral volume loss; and 3 patients had hippocampal atrophy combined with global cerebral volume loss. Long‐term structural consequences developed in all patients (n = 7) with persisting PMA and in 21 of 38 (55%) of the patients with resolved PMA.

Some patients of this cohort who underwent follow‐up MRIs and volumetric analysis have been included in the previous publication (Bosque Varela et al, 2024).^19^

## Discussion

In this prospective MRI study on a large cohort of patients with SE, we found a huge variability of timing of PMA, a risk biomarker of t2 – the time point when brain injury may occur as a consequence of SE. In humans, the time when SE‐related cerebral alterations occur depends not only on duration of SE, as suggested in the ILAE definition,^5^ but also on SE semiology, its etiology, and the patients’ level of consciousness. Based on real clinical and imaging data of 256 patients with SE, we developed a model, which may predict the risk of cerebral injury due to SE at a given time point in a patient with a distinct clinical profile. This predictive capability holds promise for individualizing the time point t2 and refining treatment approaches by carefully balancing risks of long‐term consequences of SE against potential side effects of aggressive treatments.^29^ In our cohort, SE duration was significantly longer in patients with PMA suggesting that identification of patients at high risk for developing PMA using this model could prompt earlier escalation of treatment, earlier cessation of SE, and potential improvement of outcomes.

Level of consciousness in SE is closely tied to its outcomes, with decreased consciousness linked to higher short‐term mortality.^1^, ^30^, ^31^ NCSE in coma can lead to mortality rates as high as 42%, as opposed to SE with isolated convulsive phase with regaining of consciousness (0%).^1^ Our group previously proposed a “3‐dimensional biological continuum” for NCSE, where decreased consciousness was associated with worse outcomes and higher likelihood of brain injury.^32^ In addition, NCSE in coma has also been linked to the development of new neurological deficits and long‐term mortality, as evidenced by a recent retrospective study.^33^ Some studies have attributed the association of stupor and coma to the severity of underlying etiology, resulting ultimately in the worse outcome of SE.^34^

Our findings suggest a robust correlation between the decreased level of consciousness and the probability of PMA occurrence. Patients admitted in a stuporous or comatose state may have a 5 times greater chance of developing PMA compared with alert or somnolent individuals. Intriguingly, diffusion‐restricted lesions were significantly more prominent in patients with stupor or coma, across all etiological groups, without any discernible influence of etiology. Conversely, the occurrence of FLAIR‐hyperintensity was influenced by acute etiologies.

Whereas it is well‐established that generalized convulsive SE may result in irreversible lesions based on findings from animal models^9^ and human data,^35^ the question of whether NCSE induces brain injury has been a longstanding subject of debate.^36^ The classical example is the absence SE, where brain damage has never been demonstrated in humans^37^ or in animal models,^38^ consequently, there is no clear time point t2 for absence of SE. Our data align with these findings, as none of the 5 patients with absence SE, who were excluded from the full analysis of this study, developed PMA.

We found a large heterogeneity of PMA as a risk biomarker of t2 among different SE semiologies: NCSE may have higher chances of developing PMA when compared to SE‐PM, even at 10 minutes after the onset. These findings challenge the conventional temporal threshold of 1 hour for t2 in NCSE, prompting its reevaluation. Furthermore, our results contribute to the ongoing debate on NCSE and its potential for causing brain injury, underscoring the notion that patients with NCSE may indeed be at risk of cerebral damage. On the other hand, our data also question the accepted t2‐threshold of 30 minutes for SE‐PM, which is based primarily on animal data from the 1970s.^8^ Based on our results, in this group of patients, only those who develop SE‐PM into NCSE or have acute etiologies and decreased level of consciousness (stupor or coma) have over a 50% chance of developing PMA in the form of diffusion restriction and/or FLAIR‐hyperintensity. Thus, in patients with SE‐PM, as in other cases, we suggest considering t2 based on a multimodal approach.

Etiology has been identified as a cornerstone predictor of outcome in SE.^39^ Acute primary and secondary CNS as well as acute toxic etiologies tend to be associated with worse outcomes.^40^ Consistent with these findings, we demonstrated a higher incidence of diffusion restriction and FLAIR‐hyperintensity in patients with these etiologies compared with other ones at any given time point.

Whereas this study was limited to the acute phase of SE and focused on PMA as a surrogate marker of possible cerebral injury, its long‐term implications remained a critical question. Based on the analysis of follow‐up imaging 4 weeks after the index episode of SE, we could demonstrate that over 60% of patients develop structural changes in form of either local cortical and hippocampal atrophy or global cerebral volume loss. Some of the patients who developed long‐term structural changes in the follow‐up MRI, or had their PMA resolved since the initial acute MRI, suggesting that the resolution of PMA does not prevent developing long‐term consequences and that their occurrence in the acute period of SE is indeed associated with the risk of developing long‐term consequences.

## Strengths and Limitations

We demonstrated for the first time that occurrence of PMA is rather multifactorial as it is influenced by the level of consciousness, semiology, and etiology in combination rather than by a single factor (eg, duration of SE). This innovative approach not only provides valuable insights into the factors influencing development of PMA but also enhances its relevance to clinical practice by offering a more holistic perspective. Such a comprehensive approach has the potential to significantly improve therapeutic decision making and management strategies for patients with SE.

This study also has several limitations. The exact timing of PMA occurrence could not be determined as the patients could not be imaged continuously from the beginning of SE until the appearance of PMA. Therefore, we chose as the possible time of PMA occurrence either the time point of SE cessation or the time when MRI was performed (in patients with an ongoing SE). Combining these 2 possibilities into one model introduces significant heterogeneity. We did not include into the analysis those patients with ongoing SE who did not demonstrate PMA in the acute MRI, which may have influenced the analysis.

We included SE semiology, its etiology, and the patients’ level of consciousness in our prediction model acknowledging that there might be certain collinearity among these variables.

Absence of continuous EEG in cases of NCSE necessitated estimating the SE duration based on symptomatology rather than the precise EEG measurement.

Due to different treatment regimens and medication dosages, we could not incorporate treatment into the predictive model. We acknowledge that more vigorous treatment of patients with SE‐PM or with decreased level of consciousness as opposed to those with NCSE could have influenced the occurrence of PMA.

This study included only adult patients and we cannot extrapolate our findings to children with SE. Furthermore, the presented predictive model requires external validation.

Follow‐up imaging was performed only in a subgroup of patients who demonstrated PMA on the initial MRI. Over 60% of these patients developed structural long‐term consequences. As we did not image patients without PMA, we cannot exclude that they could also have cerebral injury due to SE.

## Conclusions

This prospective MRI study reveals the complex interplay between various clinical factors and timing of occurrence of acute brain injury in MRI in patients with SE. Patients with decreased level of consciousness, NCSE, and acute etiologies may have a high risk of developing PMA at very early stages of an SE. Our model integrates semiology, level of consciousness, and etiology in predicting the occurrence of PMA – a risk biomarker of time point t2, offering clinically relevant information which might influence the management of SE.

## Author Contributions

E.T., G.K., P.B.V., G.Z., and L.M. contributed to the conception and design of the study. P.B.V., L.M., A.O., J.S., T.P., W.L., B.C.P., and J.P. participated in the acquisition and analysis of data. P.B.V., G.K., T.P., W.L., G.Z., M.C., J.P., M.L., and E.T. contributed to drafting and revising a significant portion of the manuscript, tables, and figures.

## Potential Conflict of Interest

None of the authors have potential conflict of interest directly related to this work.

## Data availability

The data supporting the findings of this study are available from the corresponding author upon reasonable request. Study datasets are not made publicly available due to ethical and data protection restrictions.

## Supporting information


Supplementary Data S1.

## References

[1] Leitinger M , Trinka E , Giovannini G , et al. Epidemiology of status epilepticus in adults: a population‐based study on incidence, causes, and outcomes. Epilepsia 2019;60:53–62. 30478910 10.1111/epi.14607 PMC7380005

[2] Trinka E , Rainer LJ , Granbichler CA , et al. Mortality, and life expectancy in epilepsy and status epilepticus‐current trends and future aspects. Front Epidemiol 2023;3:1081757. 38455899 10.3389/fepid.2023.1081757 PMC10910932

[3] Rodrigo‐Gisbert M , Abraira L , Quintana M , et al. Risk assessment of long‐term epilepsy after de novo status epilepticus with clinical and electroencephalographic biomarkers: the AFTER score. Epilepsy Behav 2023;149:109531. 37995538 10.1016/j.yebeh.2023.109531

[4] Lattanzi S , Orlandi N , Giovannini G , et al. The risk of unprovoked seizure occurrence after status epilepticus in adults. Epilepsia 2024;65:1006–1016. 38339985 10.1111/epi.17912

[5] Trinka E , Cock H , Hesdorffer D , et al. A definition and classification of status epilepticus‐‐report of the ILAE task force on classification of status epilepticus. Epilepsia 2015;56:1515–1523. 26336950 10.1111/epi.13121

[6] Lowenstein DH , Bleck T , Macdonald RL . It's time to revise the definition of status epilepticus. Epilepsia 1999;40:120–122. 9924914 10.1111/j.1528-1157.1999.tb02000.x

[7] Trinka E , Shorvon S . Status epilepticus‐‐where are we in 2013? Epilepsia 2013;54:1–2. 10.1111/epi.12263 24001059

[8] Meldrum BS , Horton RW . Physiology of status epilepticus in primates. Arch Neurol 1973;28:1–9. 4629380 10.1001/archneur.1973.00490190019001

[9] Wasterlain CG , Fujikawa DG , Penix L , Sankar R . Pathophysiological mechanisms of brain damage from status epilepticus. Epilepsia 1993;34:S37–S53. 8385002 10.1111/j.1528-1157.1993.tb05905.x

[10] Bauer G , Gotwald T , Dobesberger J , et al. Transient and permanent magnetic resonance imaging abnormalities after complex partial status epilepticus. Epilepsy Behav 2006;8:666–671. 16503204 10.1016/j.yebeh.2006.01.002

[11] Lattanzi S , Giovannini G , Brigo F , et al. Acute symptomatic status epilepticus: splitting or lumping? A proposal of classification based on real‐world data. Epilepsia 2023;64:e200–e206. 37597263 10.1111/epi.17753

[12] Bosque Varela P , Machegger L , Oellerer A , et al. Imaging of status epilepticus: making the invisible visible. A prospective study on 206 patients. Epilepsy Behav 2023;141:109130. 36803874 10.1016/j.yebeh.2023.109130

[13] Giovannini G , Kuchukhidze G , McCoy MR , et al. Neuroimaging alterations related to status epilepticus in an adult population: definition of MRI findings and clinical‐EEG correlation. Epilepsia 2018;59:120–127. 30129213 10.1111/epi.14493

[14] Bonduelle T , Ollivier M , Trin K , et al. Association of Peri‐ictal MRI abnormalities with mortality, antiseizure medication refractoriness, and morbidity in status epilepticus. Neurology 2023;100:e943–e953. 36443013 10.1212/WNL.0000000000201599 PMC9990431

[15] Hocker S , Nagarajan E , Rabinstein AA , et al. Progressive brain atrophy in super‐refractory status epilepticus. JAMA Neurol 2016;73:1201–1207. 27533350 10.1001/jamaneurol.2016.1572

[16] Mariajoseph FP , Sagar P , Muthusamy S , et al. Seizure‐induced reversible MRI abnormalities in status epilepticus: a systematic review. Seizure 2021;92:166–173. 34525432 10.1016/j.seizure.2021.09.002

[17] Cartagena AM , Young GB , Lee DH , Mirsattari SM . Reversible and irreversible cranial MRI findings associated with status epilepticus. Epilepsy Behav 2014;33:24–30. 24614522 10.1016/j.yebeh.2014.02.003

[18] Provenzale JM , Barboriak DP , VanLandingham K , et al. Hippocampal MRI signal hyperintensity after febrile status epilepticus is predictive of subsequent mesial temporal sclerosis. AJR Am J Roentgenol 2008;190:976–983. 18356445 10.2214/AJR.07.2407

[19] Bosque Varela P , Machegger L , Steinbacher J , et al. Brain damage caused by status epilepticus: a prospective MRI study. Epilepsy Behav 2024;161:110081. 39489995 10.1016/j.yebeh.2024.110081

[20] Lewis DV , Voyvodic J , Shinnar S , et al. Hippocampal sclerosis and temporal lobe epilepsy following febrile status epilepticus: the FEBSTAT study. Epilepsia 2024;65:1568–1580. 38606600 10.1111/epi.17979 PMC11166525

[21] Nairismagi J , Grohn OH , Kettunen MI , et al. Progression of brain damage after status epilepticus and its association with epileptogenesis: a quantitative MRI study in a rat model of temporal lobe epilepsy. Epilepsia 2004;45:1024–1034. 15329065 10.1111/j.0013-9580.2004.08904.x

[22] Bosque Varela P , Tabaee Damavandi P , Machegger L , et al. Magnetic resonance imaging fingerprints of status epilepticus: a case‐control study. Epilepsia 2024;65:1620–1630. 38507291 10.1111/epi.17949

[23] Chatzikonstantinou A , Gass A , Forster A , et al. Features of acute DWI abnormalities related to status epilepticus. Epilepsy Res 2011;97:45–51. 21802259 10.1016/j.eplepsyres.2011.07.002

[24] Mendes A , Sampaio L . Brain magnetic resonance in status epilepticus: a focused review. Seizure 2016;38:63–67. 27156207 10.1016/j.seizure.2016.04.007

[25] Nakae Y , Kudo Y , Yamamoto R , et al. Relationship between cortex and pulvinar abnormalities on diffusion‐weighted imaging in status epilepticus. J Neurol 2016;263:127–132. 26530510 10.1007/s00415-015-7948-4

[26] Szabo K , Poepel A , Pohlmann‐Eden B , et al. Diffusion‐weighted and perfusion MRI demonstrates parenchymal changes in complex partial status epilepticus. Brain 2005;128:1369–1376. 15743871 10.1093/brain/awh454

[27] Machegger L , Bosque Varela P , Kuchukhidze G , et al. Quantitative analysis of diffusion‐restricted lesions in a differential diagnosis of status epilepticus and acute ischemic stroke. Front Neurol 2022;13:926381. 35873780 10.3389/fneur.2022.926381 PMC9301206

[28] Meletti S , Monti G , Mirandola L , et al. Neuroimaging of status epilepticus. Epilepsia 2018;59:113–119. 30160066 10.1111/epi.14499

[29] Hocker S . Anesthetic drugs for the treatment of status epilepticus. Epilepsia 2018;59:188–192. 30159894 10.1111/epi.14498

[30] Rossetti AO , Hurwitz S , Logroscino G , Bromfield EB . Prognosis of status epilepticus: role of aetiology, age, and consciousness impairment at presentation. J Neurol Neurosurg Psychiatry 2006;77:611–615. 16614020 10.1136/jnnp.2005.080887 PMC2117456

[31] Baysal‐Kirac L , Feddersen B , Einhellig M , et al. Does semiology of status epilepticus have an impact on treatment response and outcome? Epilepsy Behav 2018;83:81–86. 29660507 10.1016/j.yebeh.2018.03.027

[32] Bauer G , Trinka E . Nonconvulsive status epilepticus and coma. Epilepsia 2010;51:177–190. 19744116 10.1111/j.1528-1167.2009.02297.x

[33] Roberg LE , Monsson O , Kristensen SB , et al. Prediction of long‐term survival after status epilepticus using the ACD score. JAMA Neurol 2022;79:604–613. 35404392 10.1001/jamaneurol.2022.0609 PMC9002715

[34] Neligan A , Shorvon SD . Prognostic factors, morbidity and mortality in tonic‐clonic status epilepticus: a review. Epilepsy Res 2011;93:1–10. 20947300 10.1016/j.eplepsyres.2010.09.003

[35] Shorvon S . Does convulsive status epilepticus (SE) result in cerebral damage or affect the course of epilepsy‐‐the epidemiological and clinical evidence? Prog Brain Res 2002;135:85–93. 12143372 10.1016/S0079-6123(02)35009-X

[36] Kaplan PW . No, some types of nonconvulsive status epilepticus cause little permanent neurologic sequelae (or: ‘the cure may be worse than the disease’). Neurophysiol Clin 2000;30:377–382. 11191930 10.1016/s0987-7053(00)00238-0

[37] Shirasaka Y . Lack of neuronal damage in atypical absence status epilepticus. Epilepsia 2002;43:1498–1501. 12460251 10.1046/j.1528-1157.2002.10502.x

[38] Wong M , Wozniak DF , Yamada KA . An animal model of generalized nonconvulsive status epilepticus: immediate characteristics and long‐term effects. Exp Neurol 2003;183:87–99. 12957492 10.1016/s0014-4886(03)00099-2

[39] Ascoli M , Ferlazzo E , Gasparini S , et al. Epidemiology and outcomes of status epilepticus. Int J Gen Med 2021;14:2965–2973. 34234526 10.2147/IJGM.S295855 PMC8254099

[40] Lattanzi S , Trinka E , Brigo F , Meletti S . Clinical scores and clusters for prediction of outcomes in status epilepticus. Epilepsy Behav 2023;140:109110. 36758360 10.1016/j.yebeh.2023.109110

