# Supplementary material for: Timing of Peri‐Ictal MRI Abnormalities in Status Epilepticus – One Size Does Not Fit All
Source: Ann Neurol. 2025 Nov 24;99(2):523–34. doi: 10.1002/ana.78076 (PMC12894505; doi:10.1002/ana.78076)
Supplement: Supplementary file 1 — Supplementary Data S1. [file ANA-99-523-s001.pdf]

Supplementary material

Supplementary Table 1: Predictive probabilities of diffusion-restricted lesions; etiology I (Acute-triggering factors associated with epilepsy (TFE))

Time points	SE-PM (alert/somnolent vs. stupor/coma)	NCSE (alert/somnolent vs. stupor/coma)	SE-PM to NCSE (alert/somnolent vs. stupor/coma)
0.16 hours	2% / 10%	10%/ 37%	4% /17%
0.33 hours	2% / 10%	10%/ 38%	4%/17%
0.50 hours	2%/ 10%	10%/ 38%	4%/17%
0.66 hours	2%/ 10%	10%/ 38%	4% /17%
0.83 hours	2%/ 10%	10%/ 38%	4%/17%
1 hour	3%/ 10%	10%/ 38%	4%/17%
1.50 hours	2%/ 10%	10%/ 38%	4%/17%
2 hours	2% / 10%	11%/ 39%	4%/18%
3 hours	2%/ 10%	11%/ 39%	4%/18%
6 hours	2%/ 11%	12%/ 41%	4%/19%
12 hours	3%/ 13%	13%/ 45%	5%/22%
24 hours	4%/ 16%	17%/ 52%	7%/27%
48 hours	6%/ 26%	28%/ 67%	11%/41%

Supplementary Table 2: Predictive probabilities of diffusion-restricted lesions; etiology II (Acute-primary CNS insult, acute secondary insults and acute-toxic)

Time points	SE-PM (alert/somnolent vs. stupor/coma)	NCSE (alert/somnolent vs. stupor/coma)	SE-PM to NCSE (alert/somnolent vs. stupor/coma)
0.16 hours	16% / 51%	52% / 86%	27% / 67%
0.33 hours	16% / 51%	53% / 86%	27% / 67%
0.50 hours	16% / 51%	53% / 86%	27% / 67%
0.66 hours	17% / 51%	53% / 86%	28% / 67%
0.83 hours	17% / 51%	53% / 86%	28% / 67%
1 hour	17% / 52%	53% / 86%	28% /67%
1.50 hours	17% / 52%	53% / 86%	28% / 68%
2 hours	17% / 52%	54% / 86%	28% / 68%
3 hours	17% / 53%	54% / 86%	29% / 68%
6 hours	18% / 55%	56% / 87%	30% / 70%
12 hours	21% / 59%	60% / 89%	34% / 73%
24 hours	26% / 66%	67% / 92%	41% /79%
48 hours	40% / 78%	79% / 95%	56% / 87%

Supplementary Table 3: Predictive probabilities of diffusion-restricted lesions; etiology III (Progressive, remote, unknown, SE in electro-clinical syndromes)

Time points	SE-PM (alert/somnolent vs. stupor/coma)	NCSE (alert/somnolent vs. stupor/coma)	SE-PM to NCSE (alert/somnolent vs. stupor/coma)
0.16 hours	8%/31%	32%/72%	14%/47%
0.33 hours	8%/31%	33%/72%	14%/47%
0.50 hours	8%/31%	33%/72%	14%/47%
0.66 hours	8%/32%	33%/72%	14%/47%
0.83 hours	8%/32%	33%/72%	14%/47%
1 hour	8%/32%	33%/72%	14%/47%
1.50 hours	8%/32%	33%/73%	14%/48%
2 hours	8%/32%	33%/73%	15%/48%
3 hours	8%/33%	34%/73%	15%/48%
6 hours	9%/35%	36%/75%	16%/50%
12 hours	10%/38%	39%/78%	18%/54%
24 hours	14%/46%	47%/82%	23%/62%
48 hours	22%/61%	62%/90%	36%/75%

Supplementary Table 4: Predictive probabilities of FLAIR-hyperintensity; etiology I (Acute-triggering factors associated with epilepsy (TFE))

Time points	SE-PM (alert/somnolent vs. stupor/coma)	NCSE (alert/somnolent vs. stupor/coma)	SE-PM to NCSE (alert/somnolent vs. stupor/coma)
0.16 hours	2%/12%	10%/37%	4%/20%
0.33 hours	2%/12%	10%/37%	4%/20%
0.50 hours	2%/12%	10%/37%	4%/20%
0.66 hours	2%/12%	10%/37%	4%/20%
0.83 hours	2%/12%	10%/38%	4%/20%
1 hour	2%/13%	10%/38%	4%/20%
1.50 hours	3%/13%	10%/38%	4%/20%
2 hours	3%/13%	10%/38%	4%/21%
3 hours	3%/13%	10%/39%	5%/21%
6 hours	3%/14%	11%/42%	5%/23%
12 hours	4%/17%	13%/46%	6%/26%
24 hours	5%/23%	19%/56%	9%/35%
48 hours	11%/40%	33%/74%	17%/54%

Supplementary Table 5: Predictive probabilities of FLAIR-hyperintensity; etiology II (Acute-primary CNS insult, acute secondary insults and acute-toxic)

Time points	SE-PM (alert/somnolent vs. stupor/coma)	NCSE (alert/somnolent vs. stupor/coma)	SE-PM to NCSE (alert/somnolent vs. stupor/coma)
0.16 hours	16%/51%	44%/81%	25%/65%
0.33 hours	16%/51%	44%/82%	25%/65%
0.50 hours	16%/51%	44%/82%	25%/65%
0.66 hours	16%/51%	44%/82%	25%/65%
0.83 hours	16%/52%	45%/82%	25%/65%
1 hour	16%/52%	45%/82%	25%/65%
1.50 hours	16%/52%	45%/82%	25%/66%
2 hours	16%/53%	45%/82%	26%/66%
3 hours	17%/53%	46%/83%	26%/67%
6 hours	18%/56%	49%/84%	28%/69%
12 hours	21%/61%	54%/87%	32%/73%
24 hours	29%/69%	63%/91%	42%/80%
48 hours	47%/83%	79%/96%	61%/90%

Supplementary Table 6: Predictive probabilities of FLAIR-hyperintensity; etiology III (Progressive, remote, unknown, SE in electro-clinical syndromes)

Time points	SE-PM (alert/somnolent vs. stupor/coma)	NCSE (alert/somnolent vs. stupor/coma)	SE-PM to NCSE (alert/somnolent vs. stupor/coma)
0.16 hours	6%/25%	20%/59%	9%/37%
0.33 hours	6%/25%	20%/59%	10%/37%
0.50 hours	6%/25%	20%/59%	10%/37%
0.66 hours	6%/25%	20%/59%	10%/37%
0.83 hours	6%/26%	21%/59%	10%/38%
1 hour	6%/26%	21%/59%	10%/38%
1.50 hours	6%/26%	21%/60%	10%/38%
2 hours	6%/26%	21%/60%	10/38%
3 hours	6%/27%	22%/61%	10%/39%
6 hours	7%/29%	23%/63%	11%/42%
12 hours	8%/33%	27%/68%	13%/46%
24 hours	12%/42%	36%/76%	19%/56%
48 hours	22%/62%	55%/87%	33%/74%

Supplementary Table 7: Absolute numbers and cumulative rate of patients with either DWI or FLAIR abnormalities in different time intervals of the SE duration

**Duration of SE**	**DWI**			**FLAIR**	
	Absolute N	%		Absolute N	%
**<10 min**	0	0		0	0
**<15 min**	0	0		0	0
**<30 min**	3	6,52		2	4,35
**<45 min**	9	13,04		7	10,14
**<60 min**	13	15,29		11	12,94
**<1 h**	27	21,43		24	19,05
**<2 h**	39	24,38		30	18,75
**<3 h**	46	25,70		37	20,67
**<4 h**	51	26,56		41	21,35
**<5 h**	59	28,23		48	22,97
**<10 h**	59	28,23		48	22,97
**<20 h**	66	30,14		55	25,11
**<30 h**	82	33,47		71	28,98
**<40 h**	83	33,60		71	28,74
**<50 h**	85	33,46		73	28,74
**<60 h**	85	33,46		73	28,74
**<80 h**	86	33,73		74	29,02
**<130 h**	87	33,98		75	29,30

Appendix 1

MRI protocol for patients with status epilepticus

DWI: spin echo-EPI diffusion imaging was used to acquire 28 slices with echo time (TE) 47 ms, repetition time (TR) 3.051 ms, field of view (FOV) 230 × 230 mm, and voxel 2.05 × 2.56 mm, with a slice thickness of 4 mm and a gap between the slices of 1 mm. The diffusion sequence was acquired with four b-values of 0, 333, 666, and 1.000 s/mm² to calculate the conventional ADC map. Diffusion gradients were applied in three directions.

FLAIR: MS TSE was used to acquire 28 slices with echo time (TE) 125 ms, repetition time (TR) 10000 ms, inversion time 2800 ms (TI), field of view (FOV) 560 mm × 560 mm, and voxel 0.65 × 1.13 mm, with a slice thickness of 4 mm and a gap between the slices of 5 mm.

T1 3D Sequence: T1W_FFE was obtained in a sagittal alignment with echo time (TE) 4.04 ms, repetition time (TR) 8.66 ms, field of view (FOV) 320 mm × 320 mm, and voxel 1 × 1 × 1 mm. T1W_FFE was done before and after administration of gadolinium.

pCASL: MS FFE single shot EPI with 39 as EPI factor. 20 slices, field of view (FOV) 240 mm x 240 mm x 119 mm, voxel 2.75 mm x 2.75 mm, with a slice thickness of 5 mm and a gap of 1 mm. Scan duration 4:28 minutes, fat saturation SPIR, temporal resolution 30 pictures, labeling distance 90, post labeling delay 1800 ms which was adapted to the age of patients. TR 4324 ms, TE 13 ms. This scan was operating in 1st level controlled mode.

T2* perfusion: Epi FFE with 40 dynamic scans had a voxel size of 2.33 x 2.33 x 4.00mm and a scan duration of 1:14. Dynamic scan time was "shortest". TR/TE was 1714/40 ms with SPIR fat suppression. Gadolinium application was started manually.

TOF: 3D FFE TOF was done in 200 slices. Scan duration 4:08. Field of view 200 x 200 x 140 mm, voxel size 0.54 x 0.82 x 1.40 mm and chunk thickness of 14 mm. TR/TE was 23/3.5 ms. One parallel suppression pulse in "head" position with a thickness of 30 mm a gap of 10 mm was used.

DTI: single shot EPI diffusion with echo time (TE) 74.43 ms, repetition time (TR) 6398.99 ms, field of view (FOV) 128 × 128 mm, and voxel 2.00 × 2.04 mm, with a slice thickness of 2 mm and a gap between the slices of 0 mm. The diffusion sequence was acquired with two b-values of 0 and 800 s/mm²; 16 spatial directions were measured.
